# Effect of accelerated electron beam on mechanical properties of human cortical bone: influence of different processing methods

**DOI:** 10.1007/s10561-012-9312-6

**Published:** 2012-05-16

**Authors:** Artur Kaminski, Ewelina Grazka, Anna Jastrzebska, Joanna Marowska, Grzegorz Gut, Artur Wojciechowski, Izabela Uhrynowska-Tyszkiewicz

**Affiliations:** 1 Department of Transplantology and Central Tissue Bank, Medical University of Warsaw, ul. Chalubinskiego 5, 02-004 Warsaw, Poland; 2 National Centre for Tissue and Cell Banking, Warsaw, Poland; 3 1st Department of Clinical Radiology, Medical University of Warsaw, Warsaw, Poland

**Keywords:** Human bone, Compact bone grafts, Electron beam irradiation, Mechanical properties, Compression test

## Abstract

Accelerated electron beam (EB) irradiation has been a sufficient method used for sterilisation of human tissue grafts for many years in a number of tissue banks. Accelerated EB, in contrast to more often used gamma photons, is a form of ionizing radiation that is characterized by lower penetration, however it is more effective in producing ionisation and to reach the same level of sterility, the exposition time of irradiated product is shorter. There are several factors, including dose and temperature of irradiation, processing conditions, as well as source of irradiation that may influence mechanical properties of a bone graft. The purpose of this study was to evaluate the effect e-beam irradiation with doses of 25 or 35 kGy, performed on dry ice or at ambient temperature, on mechanical properties of non-defatted or defatted compact bone grafts. Left and right femurs from six male cadaveric donors, aged from 46 to 54 years, were transversely cut into slices of 10 mm height, parallel to the longitudinal axis of the bone. Compact bone rings were assigned to the eight experimental groups according to the different processing method (defatted or non-defatted), as well as e-beam irradiation dose (25 or 35 kGy) and temperature conditions of irradiation (ambient temperature or dry ice). Axial compression testing was performed with a material testing machine. Results obtained for elastic and plastic regions of stress–strain curves examined by univariate analysis are described. Based on multivariate analysis, including all groups, it was found that temperature of e-beam irradiation and defatting had no consistent significant effect on evaluated mechanical parameters of compact bone rings. In contrast, irradiation with both doses significantly decreased the ultimate strain and its derivative toughness, while not affecting the ultimate stress (bone strength). As no deterioration of mechanical properties was observed in the elastic region, the reduction of the energy absorption capacity of irradiated bone rings apparently resulted from changes generated by irradiation within the plastic strain region.

## Introduction

Accelerated electron beam (EB) irradiation has been a sufficient method used for sterilisation of human tissue grafts for many years in a number of tissue banks (Dziedzic-Goclawska et al. 2005). Several studies have been carried out to introduce beam of accelerated electrons for sterilisation of particular tissue grafts, e.g. patellar tendon, using conventional—one step (Kaminski et al. 2009; Hoburg et al. 2010), or newly proposed fractionated method of radiation-sterilisation (Hoburg et al. 2011). In both types of experiments evaluated irradiation doses exceeded 30 kGy, the dose which is very often acknowledged to impair allograft biomechanical properties (Pelker et al. 1993; Cornu et al. 2000). A beam of accelerated electrons was introduced in the 1950s for radiation-sterilisation of some disposable medical devices. Due to improvement of irradiation equipment, in the 1970s EB became an acceptable method for sterilisation of a wide range of health care products including tissue grafts.

Numerous experiments has been done to study effect of irradiation on bone allografts mechanical properties. Most of them used gamma rays as an irradiation source (Komender 1976; Pelker et al. 1984; Godette et al. 1996; Currey et al. 1997; Stevenson 1999; Cornu et al. 2000). There are limited data regarding the effect of accelerated EB irradiation on biomechanical properties of banked bone allografts, especially compact bone (Hemigou et al. 1993; Dziedzic-Goclawska et al. 2005).

Accelerated EB, in contrast to gamma irradiation, is a form of ionizing radiation that is characterized by the lower penetration into the material. In consequence, the 50 % reduction of accelerated EB dose occurs in materials of 2 g/cm^3^ density (approximate compact bone density) at the depth of 1.8 cm only, whereas the same decrease is observed with gamma rays at the depth of 6 cm. Moreover, gamma irradiation, with its better penetrability inside the material, shows more uniform distribution of dose inside the graft (Kaminski et al. 2010). Due to this limitation of EB, relatively thin compact bone grafts (width up to 2 cm) can be sterilised by this technique. However, sterilisation of less dense allografts (skin, cartilage, amniotic membrane, tendons, ligaments), as well as cancellous bone or thin compact bone bars, using the one side EB irradiation seems satisfactory enough. In order to improve the homogeneity of irradiation dose inside the graft and to avoid graft size limitation for higher density grafts (compact bone, massive bone allografts), it is advisable to apply two-side radiation treatment (Hemigou et al. 1993; Dziedzic-Goclawska et al. 2005; Kaminski et al. 2010).

On the other hand, accelerated electrons are more effective than gamma photons in producing ionisation, and to reach the same level of sterility, the exposition time of irradiated product is much shorter when EB is applied as compared to gamma rays (seconds/minutes vs. several hours, respectively). Due to the short time required for sterilisation with accelerated electrons, it is much easier to control conditions of irradiation, including the temperature. The time of exposition, up to few minutes during this process, allows to keep tissue grafts in frozen state (on dry ice). However, since accelerated electrons are more effective then gamma photons, due to the thermalisation process more heat may be locally emitted inside the graft in a unit of time (Kaminski et al. 2010). If grafts are irradiated in a frozen state, the effect of temperature increase may be avoided because of the short time of exposition. The temperature increase during irradiation with accelerated electrons may play a role when grafts are sterilised at room temperature. Radiation-sterilisation alters medullary lipids of the bone graft. It was found that irradiated medullary lipids release toxic compounds for osteoblast-like cells (Moreau et al. 2000) and may alter bone healing processes. One of the reasons for this toxic effect may be the influence of the temperature raise. Defatting procedure introduced into the bone grafts processing in some tissue banks had to reduce this toxic effect.

This study evaluated the effect of accelerated electrons beam irradiation with doses of 25 or 35 kGy, performed on dry ice or at ambient temperature on mechanical properties of non-defatted or defatted compact bone grafts.

## Materials and methods

### Specimen preparation

Left and right femurs from six male cadaveric donors were harvested during multi-tissue procurement performed by tissue bank. The donor ages ranged from 46 to 54 years (mean age 51 ± 3 years). Donors were evaluated according to medical and social history, serological testing. Procured bones were processed according to standard operating procedures approved in our tissue bank in the processing laboratories in D and C air classes. Femurs were mechanically cleaned of soft tissues and stored at −70 °C. Epiphyses of the frozen femurs were cut off and stored at −70 °C for future experiments.

**Fig. 1:**
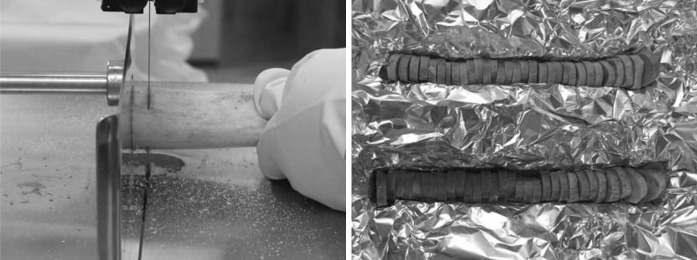
Preparing of bone rings

Isolated frozen femoral shafts were transversely cut into slices of 10 mm height, parallel to the longitudinal axis of the bone (Fig. 1), using a band saw (Model SX 220, DADAUX S.A.S, France). Each bone slice received its individual code name enabling identification of the given donor left or right femoral shaft, as well as the slice localization along the shaft length. Bone marrow was removed from femoral slices and stored frozen (−70 °C) for further lipid studies.

Femoral rings (48 pieces) obtained from six left femurs were defatted according to standard procedure approved in our tissue bank. Briefly, the procedure consisted of the following steps: (1) rinsing under shaking of thawed bone specimens in distilled water (37 °C, 5 × 5 min.); (2) defatting under shaking in 96 % ethanol with 3 % of diethylether additive (ambient temperature, 2 × 15 min.); (3) rinsing in defatting solution (ambient temperature, 1 × 5 min.); (4) passive evaporation on absorbent paper (ambient temperature, 1 × 30 min.); (5) rinsing in distilled water (4 °C, 2 × 5 min.); (6) draining off the excess of water on absorbent paper (4 °C, 1 × 10 min.).

Femoral rings (65 pieces) obtained from right femurs were not defatted.

### Experimental groups

Femoral compact bone rings from left and right bones were assigned to the eight experimental groups according to the different processing method (defatted or non-defatted), as well as EB irradiation dose (25 or 35 kGy) and temperature conditions of irradiation (ambient temperature or dry ice). Group of untreated femoral shaft rings (non-defatted and non-irradiated) served as control (Table 1).

**Table 1:** Description of the experimental and control groups

Group	N	Description
Control	18	Fresh-frozen, non-defatted, and non-irradiated control
25-EB-AT-NDF	12	Fresh-frozen, non-defatted, irradiated with 25 kGy at ambient temperatur
35-EB-AT-NDF	11	Fresh-frozen, non-defatted, irradiated with 35 kGy at ambient temperature
25-EB-DI-NDF	12	Fresh-frozen, non-defatted, irradiated with 25 kGy on dry ice
35-EB-DI-NDF	12	Fresh-frozen, non-defatted, irradiated with 35 kGy on dry ice
25-EB-AT-DF	12	Defatted, irradiated with 25 kGy at ambient temperature
35-EB-AT-DF	12	Defatted, irradiated with 35 kGy at ambient temperature
25-EB-DI-DF	12	Defatted, irradiated with 25 kGy on dry ice
35-EB-DI-DF	12	Defatted, irradiated with 35 kGy on dry ice

To provide the representation of femoral rings from different regions of femoral shafts, each experimental, as well as control group, contained compact bone rings of proximal, medial and distal part of femoral diaphyses in equal amount. Bone rings were double packed in polyester-polyethylene foil packages with 0.5 mL of saline in internal bag, appropriately labeled and stored at −70 °C.

### Electron beam (EB) irradiation

Experimental bone femoral shaft rings were EB-irradiated with two doses (25 or 35 kGy) at different temperature conditions (ambient temperature or dry ice) using an Electron Beam Accelerator (LAE-10; 10 MeV) at the Institute of Nuclear Chemistry and Technology, Warsaw, Poland. Specimens irradiated at ambient temperature were thawed and brought to room temperature before irradiation.

### Cross-sectional area measurement

To estimate compact bone cross-sectional area of the transversal plane of each bone ring, prior to mechanical testing all bone specimens were scanned by the Computed Tomography (Aquilion with multislice CT scan system, model TSX-101A, Toshiba Medical Systems Corporation, Japan) in the 1st Department of Clinical Radiology of the Medical University of Warsaw. The method of measurement was chosen due to irregular shapes of bone rings, making it impossible to calculate their cross-sectional areas from manual measurements using caliper. Specimen characteristics are shown in Table 2.

**Table 2:** Specimen characteristics

Group	N	Area (mm^2^)	Heigth (mm)
Control	18	454.89 ± 64.87	9.85 ± 0.40
25-EB-AT-NDF	12	489.08 ± 41.69	9.95 ± 0.32
35-EB-AT-NDF	11	487.64 ± 51.97	9.88 ± 0.39
25-EB-DI-NDF	12	489.33 ± 38.67	10.13 ± 0.28
35-EB-DI-NDF	12	482.83 ± 54.11	9.75 ± 0.46
25-EB-AT-DF	12	488.50 ± 51.22	9.96 ± 0.28
35-EB-AT-DF	12	486.92 ± 56.98	9.86 ± 0.18
25-EB-DI-DF	12	490.42 ± 48.95	10.04 ± 0.18
35-EB-DI-DF	12	479.58 ± 66.72	9.83 ± 0.23

Data shown as mean ± SD

To verify the accuracy of cross-sectional area measurement by CT, six regular ring-shaped plastic phantoms with different external and internal diameters, resembling those of femoral shaft rings, were prepared. External and internal diameter of each phantom was manually measured to 0.1 mm using caliper, and the cross-sectional area calculated according to the appropriate (Πr^2^) formula. Subsequently, phantoms were double packed in polyester-polyethylene foil packages (as experimental and control specimens), their cross-sectional areas estimated by CT and compared to measured manually.

### Mechanical testing

Mechanical testing of femoral bone rings for axial compression was performed at room temperature at the Warsaw University of Technology, using Material Testing Machine Z250 (Zwick/Roell, Germany) with Fmax 250 kN, cross-head speed of 1 mm/s, and actuator displacement recorded at sampling frequency of 100 Hz. Prior to mechanical testing, bone specimens were thawed and brought to room temperature.

Femoral bone rings were placed between two platens and compressed until failure. Mechanical parameters studied are shown in Table 3. Maximum load was obtained from the load-deformation curve, whereas the remaining mechanical parameters were obtained after transforming the load-deformation curve to the stress–strain curve (Turner and Burr 1993). Stress was calculated as the maximum load divided by the cross-sectional area of a specimen, strain—as the relative deformation of the specimen (the difference between its initial height and the actuator displacement), divided by the initial specimen height, and multiplied by 100 %.

**Table 3:** Mechanical parameters obtained from the compression test

Mechanical parameters	Description
Maximum load (N)	Fracture load from the load/deformation curve
Elastic limit (Pa)	Maximum stress in the elastic region (at yield point)
Young’s modulus (Pa)	Slope of the linear portion of the stress/strain curve within the elastic region
Strain in elastic region (%)	Relative deformation at the elastic limit (yield point)
Resilience (N/mm^2^)	Energy absorption at the elastic region (area under stress/strain curve at elastic region)
Ultimate strain (%)	Relative deformation at the point of failure
Ultimate stress (Strength) (Pa)	Maximum load divided by cross-sectional area of a specimen
Toughness (N/mm^2^)	Energy absorption at both elastic and plastic region, (area under stress/strain curve until the point of failure)

### Statistical methods

In the first step of the statistical analysis, univariate comparison between pairs of control and experimental groups was done. Normality of the distribution of compared parameters was tested using Shapiro–Wilk test. Next, comparison was done, using t-Student test (in the case of normal distribution of an analysed parameter) or Mann–Whitney test (in the case of non-normal distribution). Results of the univariate analysis are shown as mean ± SD with the associated *p* value.

In the second step, multivariate analyses were conducted. For each of the analysed parameter the model contained: processing method (non-defatting vs. defatting), radiation dose (25 kGy vs. non-irradiated control and 35 kGy vs. non-irradiated control), and temperature of irradiation (ambient temperature vs. dry ice). Results are presented as the model coefficient, its standard error and associated *p* value.

In all analyses, *p* value ≤0.05 was considered to be statistically significant.

## Results

### Cross-sectional area measurement

**Fig. 2:**
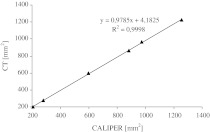
Correlation between six phantom ring cross-sectional areas estimated by CT and measured manually using CALIPER

The results of the comparison of six phantom ring cross-sectional areas estimated by CT and measured manually using caliper are shown in Table 4 and in Fig. 2. Mean percent difference in the results obtained was 1.48 ± 0.86 mm^2^ (Table 4), and measurements resulting from both methods showed strong positive linear correlation with the regression coefficient R^2^ equal to 0.9998 (Fig. 2).

**Table 4:** Comparison of phantom ring cross-sectional areas measured by CT or CALIPER

Phantom ring number	Cross-sectional area (mm^2^) CT	Cross-sectional area (mm^2^) CALIPER	(%) Difference
1	202	207	2.5
2	276	278	0.8
3	595	598	0.5
4	866	882	1.8
5	964	973	0.9
6	1,226	1,256	2.4
Mean ± SD	688.17 ± 402.96	699.00 ± 411.90	1.48 ± 0.86

### Mechanical testing

**Fig. 3:**
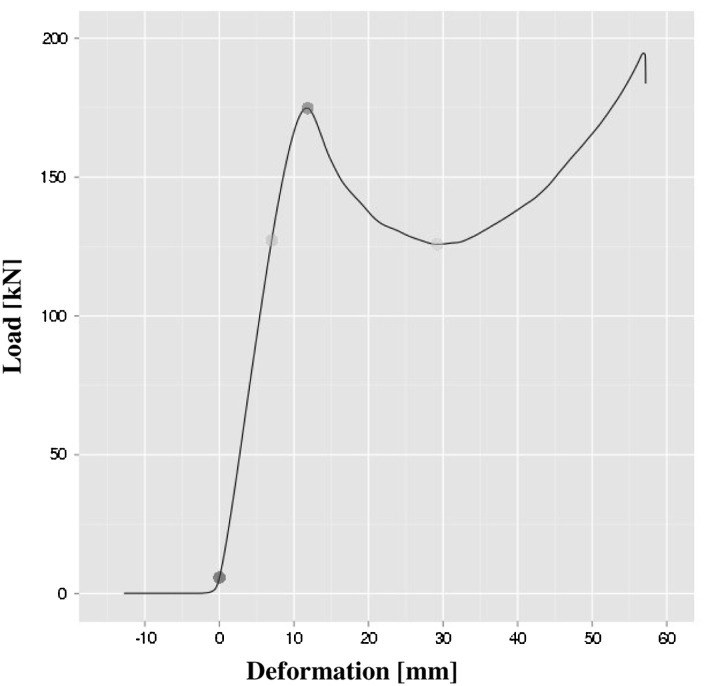
Typical load-deformation curve from the compression tests

**Fig. 4:**
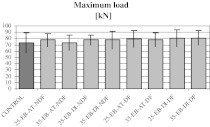
Maximum load (structural property) values from bone compression tests. No significant differences were found as compared to the control group

The typical load-deformation curve from the mechanical compression tests performed on bone rings is shown in Fig. 3. From such a curve the maximum load, being the structural mechanical property of bone rings, was obtained. As shown in Table 5 and Fig. 4, no significant differences were found in values of this parameter between experimental groups and non-defatted and non-irradiated control group.

**Table 5:** Maximum load (structural property) values from bone compression tests

Group	Maximum load (kN)
Control	72.92 ± 15.67
25-EB-AT-NDF	77.44 ± 9.92
35-EB-AT-NDF	72.61 ± 12.16
25-EB-DI-NDF	77.51 ± 8.32
35-EB-DI-NDF	77.52 ± 13.50
25-EB-AT-DF	79.59 ± 13.63
35-EB-AT-DF	77.93 ± 10.51
25-EB-DI-DF	80.74 ± 13.55
35-EB-DI-DF	80.90 ± 11.88

Data shown as mean ± SD

Following transformation of the load-deformation curve to the stress–strain curve, mechanical parameters, referring to the material properties of bone rings, were obtained.

**Fig. 5:**
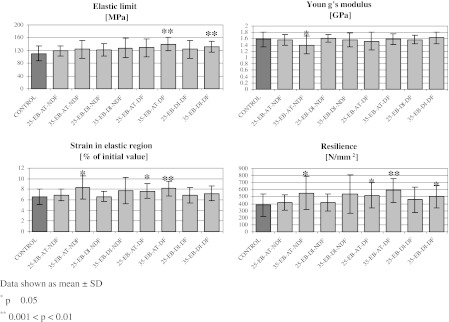
Mechanical parameters referring to the material properties of bone rings within elastic region of stress–strain curves. Significant differences are marked with *asterisks*. The level of significance is shown* below* the figure.* Data* shown as mean ± SD. *p ≤ 0.05. **0.001 < *p* < 0.01

Material properties within elastic region of the stress–strain curve are presented in Table 6 and in Fig. 5. In seven out of eight experimental groups no significant differences in Young’ modulus were observed as compared to the control one. In one non-defatted group, irradiated with the dose of 35 kGy at ambient temperature (35-EB-AT-NDF), Young’s modulus was significantly lower (−11.4 %). The increase of strain in the elastic region in this group (+26.5 %) was observed. Analysis of the elastic limit revealed the increase ranging from +7.7 to +26.1 % as compared to the control group. Statistical significance was observed in two defatted groups irradiated with the dose of 35 kGy at ambient temperature or dry ice, namely 35-EB-AT-DF (+26.1 %) and 35-EB-DI-DF (+18.7 %). Similar results were observed in the strain in the elastic region (increase range from +1.1 to +26.5 %), with three groups irradiated at ambient temperature, namely 35-EB-AT-NDF (+26.5), 25-EB-AT-DF (+17.1) and 35-EB-AT-DF (+23.9 %), showing significant increase of this parameter values. Also resilience was found to be increased in all experimental groups (increase range from +9.3 to +55.9 %) as compared to untreated control group. When respective pairs of groups were considered, differing in the irradiation dose, the mentioned above increase appeared to be dose-dependent. This increase was higher in groups irradiated with 35 kGy, with the highest values obtained when the irradiation was performed at ambient temperature (+45.5 % in 35-EB-AT-NDF group, and +55.9 % in 35-EB-AT-DF group).

**Table 6:** Mechanical parameters referring to the material properties of bone rings within elastic region of stress–strain curves

Group	Elastic limit (kN)	Young’s modulus (GPa)	Strain in elastic region (%)	Resilience (N/mm^2^)
Control	110.57 ± 22.56	1.58 ± 0.24	6.56 ± 1.49	379.29 ± 156.13
25-EB-AT-NDF	118.50 ± 16.30	1.56 ± 0.18	6.91 ± 1.09	417.32 ± 107.70
35-EB-AT-NDF	123.60 ± 27.60	1.40 ± 0.28*	8.30 ± 2.21*	551.99 ± 235.03*
25-EB-DI-NDF	121.00 ± 19.50	1.61 ± 0.12	6.63 ± 1.00	414.70 ± 119.36
35-EB-DI-NDF	127.90 ± 29.80	1.57 ± 0.22	7.74 ± 2.45	538.38 ± 272.67
25-EB-AT-DF	128.34 ± 27.82	1.52 ± 0.28	7.68 ± 1.39*	520.42 ± 183.45*
35-EB-AT-DF	139.38 ± 20.47**	1.58 ± 0.17	8.13 ± 1.44**	591.15 ± 170.70**
25-EB-DI-DF	123.99 ± 27.86	1.57 ± 0.14	6.90 ± 1.44	457.47 ± 183.78
35-EB-DI-DF	131.24 ± 17.76**	1.62 ± 0.18	7.22 ± 1.44	500.91 ± 155.54*

Significant differences are marked with asterisks. The level of significance is shown below the table

Data shown as mean ± SD

* *p* ≤ 0.05

** 0.001 < *p* < 0.01

**Fig. 6:**
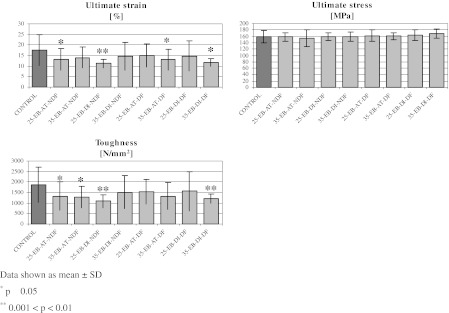
Mechanical parameters referring to the material properties of bone rings within both elastic and plastic region of stress–strain curves. Significant differences are marked with *asterisks*. The level of significance is shown* below* the figure.* Data* shown as mean ± SD. *p ≤ 0.05. **0.001 < *p* < 0.01

Mechanical parameters referring to the material properties of bone rings within both elastic and plastic regions of stress–strain curve are presented in Table 7 and in Fig. 6.

**Table 7:** Mechanical parameters referring to the material properties of bone rings within both elastic and plastic regions of stress–strain curves

Group	Ultimate strain (%)	Ultimate stress (MPa)	Toughness (N/mm^2^)
Control	17.61 ± 7.24	159.33 ± 20.15	1,859.59 ± 846.01
25-EB-AT-NDF	13.19 ± 5.15*	158.33 ± 13.25	1,326.27 ± 676.07*
35-EB-AT-NDF	14.04 ± 4.88	153.45 ± 26.50	1,280.99 ± 524.67*
25-EB-DI-NDF	11.17 ± 2.09**	159.50 ± 11.31	1,079.73 ± 296.61**
35-EB-DI-NDF	14.75 ± 6.59	159.92 ± 14.25	1,514.11 ± 782.20
25-EB-AT-DF	14.95 ± 5.48	162.42 ± 18.24	1,525.51 ± 584.23
35-EB-AT-DF	13.04 ± 4.86*	160.33 ± 11.63	1,317.81 ± 640.13
25-EB-DI-DF	14.80 ± 7.22	164.08 ± 17.81	1,562.32 ± 928.42
35-EB-DI-DF	11.76 ± 1.88*	168.67 ± 13.32	1,211.16 ± 233.35**

Significant differences are marked with asterisks. The level of significance is shown below the table

Data shown as mean ± SD

* *p* ≤ 0.05

** 0.001 < *p* < 0.01

The toughness, representing the whole area under the stress–strain curve at the point of fracture, showed the tendency towards lower values in all experimental groups, with the decreases ranging from −16.0 to −41.9 % as compared to the control group. Significant differences were observed in four groups: in two non-defatted and irradiated with both doses at ambient temperature, namely 25-EB-AT-NDF (−28.7 %) and 35-EB-AT-NDF (−31.11 %), one non-defatted and irradiated on dry ice with 25 kGy (−41.9 %; 25-EB-DI-NDF group), and one defatted and irradiated on dry ice with 35 kGy (−34.9 %; 35-EB-DI-DF group). In all experimental groups no changes were found according to irradiation dose, temperature and defatting procedure. The decrease in toughness resulted from diminished ultimate strain only, as ultimate stress (bone strength) remained unchanged in all experimental groups, irrespectively of the processing methods and irradiated doses (Fig. 5 and Table 6).

According to multivariate analysis of all groups (Table 8), it was found that temperature of EB irradiation and defatting had no significant effect on evaluated mechanical parameters of compact bone rings. Irradiation with both doses significantly decreased the ultimate strain and toughness. No significant differences were found in values of ultimate stress between experimental groups and non-defatted and non-irradiated control.

**Table 8:** Results of multivariate analysis including all groups and all mechanical parameters studied

	Maximum load	Young’s modulus	Elastic limit	Strain in elastic region	Resilience	Ultimate stress	Ultimate strain	Toughness
Dose: 25 kGy	NS	NS	NS	NS	NS	NS	↓ *p* = 0.010	↓ *p* = 0.012
Dose: 35 kGy	NS	NS	↑ *p* = 0.026	NS	↑ *p* = 0.030	NS	↓ *p* = 0.008	↓ *p* = 0.010
Ambient temperature	NS	NS	NS	NS	NS	NS	NS	NS
Defatting	NS	NS	NS	NS	NS	NS	NS	NS

*NS* *p* > 0.05

## Discussion

It has been demonstrated previously that accelerated EB irradiation is able to inactivate microorganisms at least to the same extent as gamma rays (DeLara et al. 2002; Preuss et al. 1997). Most of experimental data regarding the effect of irradiation on biological, physical and biochemical properties of bone allografts refers to gamma irradiation (Anderson et al. 1992; Godette et al. 1996; Salehpour et al. 1995; Stevenson 1999).

In our studies we were comparing mechanical properties of compact bone rings processed with or without defatting procedure and subsequently irradiated with different doses of accelerated electrons at different temperatures.

There is a number of published studies on mechanical properties of irradiated compact bone. However, as they have not been standardized, the comparison of their results is difficult or even impossible. One of the problems results from the different methods of material sampling used for the experiments by different authors. Hamer et al. (1995) suggested to use transverse sections of the femoral shaft roughly teardrop-shaped. The use of thin transverse sections of midfemoral shaft provides samples which vary little in shape, dimensions, or structure. In our studies it was decided to use cortical bone rings from whole femur diaphyses similarly as during processing of compact bone allografts in a tissue bank. To provide adequate representation of femoral rings from different diaphyseal regions, for each experimental and control group proximal, medial and distal part of femoral diaphyses in equal amount were used. Cross-sectional areas and heights of all bone samples were measured.

No statistically significant differences in maximum load, describing structural properties of compact bone rings, were observed in all experimental groups in comparison with the control one. Our results are supported by a limited number of publications, but with the use of gamma rays as an irradiation source. High doses of irradiation, up to 50 kGy, were reported not to alter significantly biomechanical characteristics of bone (Anderson et al. 1992; Tosello 1995; Dziedzic-Goclawska et al. 2005). However, in most of publications the decrease of maximum load of cortical bone was observed after gamma irradiation with doses over 30 kGy (Komender 1976; Voggenreiter et al. 1994; Godette et al. 1996; Currey et al. 1997; Stevenson 1999).

Moreover, we did not observe the influence of EB irradiation temperature (ambient vs. dry ice) on the maximum load sustained by cortical bone rings, whereas Hamer et al. (1999), using gamma irradiation with a dose of 30.2 kGy, found significantly lower values of this parameter when bone samples were irradiated without freezing. This discrepancy in the results obtained is not clear, but the type of irradiation applied in our experiment may play a key role in this respect. Additionally, methodological issues, resulting from the lack of standardisation, might contribute to those conflicting results.

The analysis of the elastic region of the stress/strain curves showed in seven out of eight experimental groups no significant differences in Young’ modulus (intrinsic stiffness of the material) as compared to controls. Similar observations regarding no effect on elastic modulus were reported by other authors even at irradiation with a dose of 60 kGy (Hamer et al. 1996; Currey et al. 1997). Only in one non-defatted group, irradiated with the dose of 35 kGy at ambient temperature, Young’s modulus was significantly lower, probably because of the marked increase of strain in the elastic region in this group, and, in consequence, change in the slope of the stress–strain curve.

Analysis of other parameters calculated from the stress/strain curves describing elastic properties of tested bone rings was based on evaluation of the elastic limit, strain in elastic region and resilience. Analysis of elastic limit revealed the tendency towards higher values as compared to the control group. Statistical significance was observed in two defatted groups irradiated with the dose of 35 kGy at ambient temperature or on dry ice. Similar tendency was observed in the strain in the elastic region, with three groups (one non-defatted and two defatted) irradiated at ambient temperature showing significant increase of this parameter values. As a result of the observed tendencies towards higher values of the elastic limit and strain in the elastic region, also resilience (energy absorption in the elastic region) was found to be increased in all experimental groups as compared to untreated control group. When respective pairs of groups were viewed, differing in the irradiation dose only, that increase appeared to be dose-dependent as it was consistently higher in groups irradiated with 35 kGy. The highest values were observed when the irradiation was performed at ambient temperature.

Opposite to the results obtained for the resilience in the elastic region, the toughness, representing the whole area under the stress–strain curve at the point of fracture (total energy absorption capacity of bone material), showed the tendency towards lower values in all experimental groups as compared to the control one. Significant differences were observed in both non-defatted and irradiated at ambient temperature groups, one non-defatted and irradiated on dry ice with 25 kGy and one defatted and irradiated on dry ice with 35 kGy. No consistent effects according to irradiation dose and temperature, as well as defatting procedure, were found. Unexpectedly, that decrease in toughness resulted from diminished ultimate strain only, as ultimate stress (bone strength) remained unchanged in all experimental groups, irrespectively of the processing methods and irradiation doses. Moreover, as the strain in the elastic region was not decreased, but even increased in the majority of experimental groups, the observed phenomenon was apparently the result of marked decrease in strain in the plastic region only, although those regions were not analyzed separately.

It was described by Burstein et al. (1975) that the plasticity of bone depends on the structure of collagen fibres. Damage of collagen fibres, as cutting of its molecules and changes in collagen inter- and intra-molecular crosslinks related to irradiation, may be responsible for the loss of mechanical properties. This finding was also described by other authors (Bowes and Moss 1962; Bailey 1968; Bright and Burstein 1978; Dziedzic-Goclawska 2000). Additionally, Hamer et al. (1996) have shown that irradiation has a dose-dependent effect on the plastic properties of bone grafts and that low temperatures prevent collagen damage during irradiation. He has studied the effect of gamma irradiation on cortical bone mechanical properties.

Although in our experiment the values of maximum load and ultimate stress (bone strength) in all experimental groups irradiated with accelerated electrons in different conditions were not affected, the changes were found in other parameters describing elastic and plastic properties of cortical bone grafts. Decreases found in analysed material properties at both elastic and plastic regions were not consistently temperature dependent and co-existed with the increases in analysed properties at the elastic region. Our results stands in contrary to previously described synergetic effect of gamma irradiation and room temperature to cause mechanical loss in the cortical bone in all parameters (Zhou et al. 2011).

It has to be taken into consideration that free radicals induced during irradiation are responsible for simultaneous scission of collagen molecules by direct effect (Cheung et al. 1990; Hamer et al. 1999) and in the same time for creation of new immature collagen crosslinks by indirect effect (Salehpour et al. 1995; Dziedzic-Goclawska et al. 2005). The impact of these processes on the final effects may differ depending on irradiation conditions (dose, temperature), physical state of a sample (Dziedzic-Goclawska et al. 2005) and a type of irradiation source used.

Despite much shorter duration, the time of irradiation with accelerated electrons plays more important role than during gamma irradiation with regard to thermalisation process. Accelerated EB irradiation at ambient temperature emits more heat inside the graft in a unit of time (Kaminski et al. 2010) whereas during e-beam irradiation in a frozen state, the effect of temperature increase may be avoided due to the short time of exposition. Therefore, EB irradiation at low temperatures both immobilises water particles and shorten the time for creation of free radicals due to the short time of exposition.

During EB irradiation at room temperature the direct effect is more efficient, but may be to some extent compensated by indirect effect. It is possible to speculate, therefore, that such phenomenon is responsible for no change of maximum load and ultimate stress observed in our experiment.

Based on multivariate analysis, it was found that temperature of e-beam irradiation and defatting procedure had no consistent significant effect on evaluated mechanical properties of compact bone rings. In contrast, irradiation with both doses significantly decreased the ultimate strain and its derivative toughness, while not affecting the ultimate stress (bone strength). As no deterioration of mechanical properties was observed in the elastic region, the reduction of the energy absorption capacity of irradiated bone rings apparently resulted from changes generated by irradiation within the plastic strain region.

## Abbreviations

EB: Electron beam
G: Gamma
CT: Computed tomography
AT: Ambient temperature
DI: Dry ice
DF: Defatted
NDF: Non-defatted
